# The association of retinal vessel calibre, white matter hyperintensities and cognitive decline in community-dwelling older adults

**DOI:** 10.1093/ageing/afaf243

**Published:** 2025-09-10

**Authors:** Catherine Robb, Amy Brodtmann, Robyn L Woods, Ruth E Trevaks, Stephanie A Ward, Meng Law, Suzanne G Orchard, Anne M Murray, Rory Wolfe, Joanne Ryan, Nigel P Stocks, Noni Rupasinghe, Raj C Shah, Christopher M Reid, Walter P Abhayaratna, Gary F Egan, Mohamed Salah Khlif, Trevor T J Chong, Liubov Robman, John J McNeil

**Affiliations:** Industrial Transformation Training Centre for Optimal Ageing, Monash University, Melbourne, Victoria, Australia; School of Psychological Sciences, Monash University, Melbourne, Victoria, Australia; School of Public Health and Preventive Medicine, Monash University, Melbourne, Victoria, Australia; Department of Neuroscience, School of Translational Medicine, Monash, Monash University, Melbourne, Victoria, Australia; School of Public Health and Preventive Medicine, Monash University, Melbourne, Victoria, Australia; School of Public Health and Preventive Medicine, Monash University, Melbourne, Victoria, Australia; Centre for Healthy Brain Ageing (CHeBA), School of Psychiatry, University of New South Wales, Sydney, New South Wales, Australia; Department of Geriatric Medicine, Prince of Wales Hospital and Community Health Services, Randwick, New South Wales, Australia; Department of Neuroscience, Central Clinical School, Monash University, Melbourne, Victoria, Australia; School of Public Health and Preventive Medicine, Monash University, Melbourne, Victoria, Australia; Geriatrics Division, Department of Medicine, Hennepin Healthcare, and Berman Center for Outcomes and Clinical Research, Hennepin Healthcare Research Institute, Minneapolis, MN, USA; Department of Epidemiology and Preventive Medicine, Monash University, Melbourne, Victoria, Australia; School of Public Health & Preventive Medicine, ASPREE, Monash University, Level 5, Alfred Centre 99 Commercial Road Prahran, Melbourne, Victoria 3004, Australia; INSERM, University Montpellier, Paris, Île-de-France, France; Adelaide Medical School, The University of Adelaide Discipline of General Practice, Adelaide, South Australia, Australia; School of Public Health and Preventive Medicine, Monash University, Melbourne, Victoria, Australia; Department of Family and Preventive Medicine and the Rush Alzheimer’s Disease Center, Rush University Medical Center, Chicago, Illinois, USA; School of Public Health and Preventive Medicine, Monash University, Melbourne, Victoria, Australia; School of Population Health, Curtin University, Perth, Australia; School of Medicine and Psychology, Australian National University, Canberra, Australian Capital Territory, Australia; Monash Biomedical Imaging, Monash University, Melbourne, Victoria, Australia; Department of Neuroscience, School of Translational Medicine, Monash, Monash University, Melbourne, Victoria, Australia; Turner Institute for Brain and Mental Health, School of Psychological Sciences, Monash University, Clayton, Victoria, Australia; Department of Neurology, Alfred Health, Melbourne, Victoria, Australia; Department of Clinical Neurosciences, St Vincent’s Hospital, Melbourne, Victoria, Australia; School of Public Health and Preventive Medicine, Monash University, Melbourne, Victoria, Australia; Centre for Eye Research Australia, The University of Melbourne, Melbourne, Victoria, Australia; Department of Epidemiology and Preventive Medicine, Monash University, Melbourne, Victoria, Australia

**Keywords:** cognition, microvasculature, ageing, retinal vessel calibre, white matter hyperintensities, older people

## Abstract

**Background:**

Retinal vessel calibres (RVCs) are non-invasive markers of microvascular health and may serve as accessible indicators of cerebral small vessel disease (CSVD) and future cognitive impairment. This study examines whether RVCs are associated with cognitive decline, and how these associations compare with those observed for white matter hyperintensities (WMH), a known marker of CSVD.

**Methods:**

Data were analysed from community-dwelling participants aged 70+ in the ASPREE trial and sub-studies, free of dementia and cardiovascular disease at baseline. RVCs were measured from fundus photography and WMH volumes from 3 T magnetic resonance imaging. Covariate-adjusted linear mixed-effects models assessed cognitive trajectories relative to baseline RVCs and WMH volumes. Cross-sectional associations between baseline RVCs and WMHs were examined via linear regression.

**Results:**

This study included 3540 participants with RVC data and 489 with WMH data (median [IQR] age: 73.2 [71.4-76.3] and 72.5 [71.2-75.4] years; female: 52.9% and 47.6%) over a median follow-up of 7.4 [IQR 5.5–8.5] and 3.8 [IQR 2.9-5.3] years, respectively. Baseline RVCs were not significantly associated with cognitive trajectories nor with baseline WMHs. Larger baseline WMH volumes were associated with greater global (Modified Mini-Mental State Examination) decline (mean 0.40 points/year; 95% CI 0.57, 0.22) and declines in delayed memory (HVLT-r) (−0.13 [−0.22, −0.04]), psychomotor function (Symbol Digit Modalities Test) (−0.29 [−0.52, −0.07]) and to a lesser extent, executive function (Controlled Oral Word Association Test) (−0.09 [95% CI −0.22, 0.03]).

**Conclusion:**

In contrast to WMH volumes, RVCs were not associated with cognitive decline. Exploring longitudinal changes in a broader range of retinal and brain biomarkers may provide deeper insights into the relationship between ocular and cerebral biomarkers in CSVD and clinical outcomes.

## Key Points

- White matter hyperintensities (WMH), but not retinal vessel calibres (RVCs), predict cognitive decline in older adults.
- Larger WMH volumes are linked to declines in global cognition, memory and psychomotor function.
- RVCs lack prognostic value for cognitive decline, unlike established cerebral small vessel disease markers.

## Introduction

Cerebral small vessel disease (CSVD) is a major contributor to dementia [1], often manifesting on brain magnetic resonance imaging (MRI) as white matter hyperintensities (WMH), a known risk factor for cognitive decline and dementia [2–4]. Given the burden of dementia and population ageing, non-invasive and cost-effective risk stratification methods are needed for timely prevention [5].

The retinal vasculature provides a unique window into the brain’s microvasculature, as retinal blood vessels are anatomically and physiologically similar to cerebral vessels and can be directly visualised in vivo [6, 7]. Retinal vessel calibre (RVC), assessed via fundus photography, represents the dimensions of retinal arterioles and venules. A hypothesised mechanism linking RVC to WMH and cognitive decline lies in their shared vascular origins: reflecting microvascular dysfunction, including endothelial damage, impaired autoregulation and blood–brain barrier breakdown [8]. These processes may contribute to chronic cerebral hypoperfusion and subsequent white matter damage, which is implicated in cognitive impairment.

Several prospective observational studies have demonstrated a positive association between RVC and incident dementia [9–11], but findings related to cognitive decline have been inconsistent (Appendix Table S1). Studies investigating the prospective association between RVC and cognition have either utilised a memory clinic population [12] middle-aged cohorts [13, 14] or individuals with pre-existing comorbidities, including cardiovascular disease [15]. This highlights a gap in understanding whether RVC relates to cognitive trajectories in healthier, older populations; a rapidly growing demographic.

The potential of non-invasive retinal imaging to facilitate early risk stratification for cognitive decline and dementia is controversial and requires more definitive evidence. This is the first study to investigate whether baseline RVC is associated with cognitive trajectories in comparison to WMH volumes, a recognised marker of CSVD. The ASPirin in Reducing Events in the Elderly (ASPREE) study [16, 17], a large cohort of initially healthy community-dwelling older adults with retinal and MRI imaging data and 11-years of cognitive follow-up data, provides a unique opportunity to examine these associations.

## Methods

### Study population

Data were from Australian ASPREE trial (2010-2017) [17] and ASPREE-XTension follow-up participants(2018-2024) [16] who also participated in retinal and brain imaging sub-studies [16, 18–21]. Further details on the study population and eligibility can be viewed in Appendix 1.1. The ASPREE trial (NCT01038583) was registered on clinicaltrials.gov.

### Retinal image measurement and analysis

Fundus imaging followed a consistent protocol between the sub-studies (Appendix 1.2) [19, 22]. Participants underwent fundus photography using non-mydriatic fundus cameras (Canon Inc., Tokyo, Japan) with a 45-degree field of view. Semi-automated Interactive Vessel Analyser (IVAN, University of Wisconsin-Madison, Ferrier NJ) software, initially developed for the (Atherosclerosis Risk in Communities) ARIC study [23], measured retinal vessel diameters in microns (μm). The diameters were combined into central retinal arteriolar equivalent (CRAE) and central retinal venular equivalent (CRVE) measures using the Parr-Hubbard ‘big six’ formula [23, 24].

### White matter hyperintensity volume measurement and analysis

MRIs were performed on a subset of participants at the Monash Biomedical Imaging facility in Melbourne, Australia, using a 3.0 Tesla Skyra scanner (Siemens, Erlangen, Germany) equipped with a 32-channel head coil. Standardised imaging sequences were employed to assess brain morphometry, microstructure and function [21]. WMH volumes were estimated using an automated lesion prediction algorithm which was manually checked and edited for accuracy. Further methodological details can be viewed in Appendix 1.3 [21].

### Cognitive assessments

Global cognitive performance was assessed using the Modified Mini-Mental State Examination (3MS) (score 0–100) [25], which served as the primary outcome. After the COVID-19 outbreak, participants completed a validated telephone adaptation of the 3MS [26]. Recognising that vascular-related cognitive changes may initially manifest in measures relating to executive function in healthy older adults [27], we included the Controlled Oral Word Association Test (COWAT—single letter version) (score 0–110) [28] and the Symbol Digit Modalities Test (SDMT) (score 0–133) [29]. Additionally, the Hopkins Verbal Learning Test-Revised (HVLT-R) delayed recall [30] (score 0-12), a measure of delayed memory, associated with early stages of Alzheimer’s disease (AD) and WMH burden [27], was included. Lower scores across all tests indicate poorer cognitive performance. To account for potential ceiling effects in this healthier population and broadening the range of detectable cognitive changes, composite z-scores were calculated to represent global cognitive performance [31], delayed memory and executive function as additional exploratory outcomes. Further methodological details are reported in Appendix 1.4.

### Statistical analyses

Baseline characteristics were summarised using descriptive statistics and stratified by CRAE, CRVE and WMH tertiles. Associations between RVCs (CRAE and CRVE), WMH volumes and cognitive performance were analysed both as continuous (z-scores) and categorical (tertiles) versions of each exposure variable, with the lowest-risk tertile serving as the reference group. This approach aligns with prior work in the field [12]. Given the skewed distribution of WMH volumes, a log transformation was applied to approximate normality for regression modelling (Appendix Figure S1).

Linear mixed-effects models (LMM) examined the association between CRAE, CRVE and WMHs and raw cognitive score trajectories (Appendix Table S2). Time points with fewer than 100 participants (i.e. year 2) were excluded. All LMMs included a continuous variable for the year since baseline (coded as 0 [baseline], 1, 3, 4, etc) and an exposure-by-year interaction to estimate whether cognitive decline rates differed by baseline retinal or WMH status. Random intercepts and slopes were specified at the participant level to account for individual differences in baseline cognition and change over time.

Models were adjusted for baseline covariates in two steps: Model 1 included age, sex and education; Model 2 added estimated glomerular filtrations rate (eGFR) and vascular risk factors. The definition and derivation of these covariates are provided in Appendix 1.5. WMH volumes were routinely adjusted for total brain volume (minus ventricular volumes). Analyses were not stratified by aspirin treatment group due to no significant effect of aspirin treatment on cognitive decline nor dementia in the ASPREE cohort [31]. Analyses were restricted to participants with at least three follow-up visits to ensure robust estimation of trajectories [32, 33].

In addition to the primary analysis of raw cognitive scores, results were also examined for composite z-scores of global cognition, executive function and memory as additional secondary outcomes. Further analysis included a cross-sectional linear regression model examining the association between baseline RVC and WMH z-scores (fully adjusted).

### Sensitivity analysis

To assess the robustness of the primary findings, several sensitivity analyses were conducted. A third model included adjustment for *APOE*ɛ4 carrier status (N = 3312), given its known association with cognitive decline. A fourth model excluded participants who completed phone-based 3MS assessments to minimise potential measurement variability due to mode of administration. To address potential bias from non-random attrition, we conducted a complete case analysis restricted to participants with follow-up data available up to year 7, after which drop-out rates increased. Additionally, we re-ran the primary models including all eligible participants regardless of the number of follow-up visits, to examine the potential impact of survivor bias introduced by our criteria of ≥3 follow-up assessments. To assess selection bias due to differences between the RVC and WMH sub-samples, we applied inverse probability weighting (IPW), reweighting the RVC sample to match the WMH sample on age, sex, education, diabetes, hypertension and dyslipidaemia. These sensitivity analyses were structured identically to the main models and included the same fixed effects and exposure-by-time interaction terms.

Regression diagnostics, including residuals, Q-Q plots and scale-location plots, were performed to confirm model assumptions and met. All analyses were conducted using Stata version 17.

## Results

### Study participants

Of the 16,703 Australian ASPREE participants, 4115 had RVC and cognitive measures. A total of 551 participants had baseline WMH data and cognitive measures; of these, 453 also had retinal imaging, while 98 did not. After excluding participants with fewer than three follow-up visits, final samples were 3540 for the retinal sub-set and 489 for the 3 T-MRI WMH sub-set (Appendix Figure S2). The median follow-up for cognitive data was 7.4 years (interquartile range [IQR] 5.5–8.5) for the retinal sub-set and 3.8 years (IQR 2.9–5.3) for the 3 T-MRI WMH sub-set.

Table 1 presents participant characteristics stratified by CRAE and CRVE tertiles, while Table 2 compares participants by WMH tertiles. Baseline characteristics in the overall ASPREE cohort, WMH sub-set and the RVC sub-sets can be viewed in Appendix 2, Table S3. The RVC sub-set consisted of 53% female participants with a median age of 73.2 years (IQR 71.4–76.3). CRAE, CRVE and WMH volumes were similar to those reported in the Multi-Ethnic Study of Atherosclerosis (MESA), a reasonably comparable middle-aged to older population, notably free of cardiovascular disease at baseline [14, 34].

**Table 1:** Characteristics of the study population according to retinal vessel calibre tertiles

	**CRAE**			**CRVE**		
	**Tertile 1** N = 1180 Mean (SD) μm = 125.9 (8.5)	**Tertile 2** N = 1181 Mean (SD) μm = 142.4 (3.6)	**Tertile 3** N = 1179 Mean (SD) μm = 158.7 (8.8)	**Tertile 1** N = 1180 Mean (SD) μm = 184.7 (11.2)	**Tertile 2** N = 1182 Mean (SD) μm = 208.2 (5.3)	**Tertile 3** N = 1178 Mean (SD) μm = 232.1 (12.8)
Age (years), median (IQR)	73.7 (71.6-77.1)	73.5 (71.5-76.2)	72.7 (71.2-75.5)	73.8 (71.6-77.2)	73.3 (71.4-76.0)	72.8 (71.3-75.5)
Female, n (%)	589 (49.9)	589 (49.9)	694 (58.9)	656 (55.6)	613 (51.9)	603 (51.2)
Education, n (% <12 years)	466 (39.5)	514 (43.5)	535 (45.4)	478 (40.5)	512 (43.3)	525 (44.6)
Smoker n (% former/current)	494 (41.9)	515 (43.6)	505 (42.8)	458 (38.8)	477 (40.4)	579 (49.2)
BMI (kg/m^2^), mean (SD)^a^	27.9 (4.4)	28.1 (4.5)	28.3 (4.7)	27.6 (4.3)	28.1 (4.5)	28.6 (4.6)
eGFR (mL/min/1.73m^2^), median (IQR)^a^	74.3 (63.9-84.0)	75.7 (65.8-85.2)	77.2 (66.7-86.3)	75.1 (64.8-84.7)	75.0 (65.3-84.5)	76.9 (66.3-86.3)
Hypertension, n (%)	896 (75.9)	877 (74.3)	784 (66.5)	848 (71.9)	863 (73.0)	846 (71.8)
Diabetes, n (%)	99 (8.4)	107 (9.1)	136 (11.5)	97 (8.2)	114 (9.6)	131 (11.1)
Dyslipidaemia, n (%)	769 (65.2)	774 (65.5)	791 (67.1)	773 (65.5)	783 (66.2)	778 (66.0)
*APOE*ɛ4 carrier^a^, n (%)	269 (24.3)	261 (23.6)	283 (25.8)	287 (25.9)	239 (21.7)	287 (26.0)
WMH (mm^3^), median (IQR)	3373.0 (2023.0-6337.0)	3295.0 (1756.0-6936.0)	3045.5 (1888.0-4910.0)	3171.5 (2005.0-5581.0)	3260.0 (1991.5-5900.5)	3126.5 (1694.0-6305.0)
3MS, mean (SD)	94.0 (4.3)	93.8 (4.2)	94.1 (4.2)	94.1 (4.2)	93.8 (4.4)	94.0 (4.2)
HVLT-R, mean (SD)	8.0 (2.7)	8.0 (2.7)	8.1 (2.7)	8.05 (2.7)	8.06 (2.8)	8.03 (2.7)
SDMT, mean (SD)	38.3 (9.3)	38.8 (9.4)	38.8 (9.3)	38.8 (9.5)	38.5 (9.6)	38.6 (9.0)
COWAT, mean (SD)	12.4 (4.7)	12.4 (4.6)	12.4 (4.7)	12.4 (4.7)	12.3 (4.7)	12.5 (4.6)

Abbreviations: IQR, interquartile range; BMI, body mass index; eGFR, estimated glomerular filtration rate; *APOE*ɛ4, apolipoprotein epsilon-4; CRAE, central retinal arteriolar equivalents; CRVE, central retinal venular equivalents; WMH, White Matter Hyperintensities; 3MS, Modified Mini-Mental State Examination; HVLT-R, Hopkins Verbal Learning Test Revised; SDMT, Symbol Digit Modalities Test; COWAT, Controlled Oral Word Association Task.

^a^% missing: BMI, 0.3%; non-HDL, 2.8%; eGFR, 2.5%; *APOE*ɛ4 carrier status, 6.9%

**Table 2:** Characteristics of the study population according to white matter hyperintensity volume tertiles

	**Total White Matter Hyperintensity Volumes (mm** ^**3**^**)**		
	Tertile 1 N = 163 Median (IQR) mm^3^ = 1463 (1075, 1872)	Tertile 2 N = 163 Median (IQR) mm^3^ = 3224 (2700, 3847)	Tertile 3 N = 163 Median (IQR) mm^3^ = 8009 (5797, 12,366)
Age (years), median (IQR)	72.1 (71.1-74.1)	72.1 (71.2-74.7)	73.9 (71.4-77.6)
Female, n (%)	79 (48.5)	84 (51.5)	70 (42.9)
Education, n (% <12 years)	69 (42.3)	49 (30.1)	68 (41.7)
Smoker n (% former/current)	60 (36.8)	70 (42.9)	77 (47.2)
BMI (kg/m2), mean (SD)^a^	27.9 (4.0)	27.9 (4.1)	27.9 (4.9)
eGFR (mL/min/1.73m^2^), median (IQR)^a^	80.6 (69.5-86.5)	76.4 (68.1-87.3)	74.7 (63.5-84.2)
Hypertension, n (%)	108 (66.3)	112 (68.7)	127 (77.9)
Diabetes, n (%)	18 (11.0)	15 (9.2)	24 (14.7)
Dyslipidaemia, n (%)	99 (60.7)	95 (58.3)	89 (54.6)
*APOE*ɛ4 carrier, n (%)^a^	35 (22.9)	34 (21.7)	48 (30.6)
CRAE (μm), mean (SD)	146.6 (14.9)	145.5 (15.3)	145.5 (16.9)
CRVE (μm), mean (SD)	213.9 (22.4)	210.0 (22.4)	213.5 (21.4)
3MS, mean (SD)	94.0 (4.6)	94.2 (4.1)	93.8 (4.0)
HVLT-R (delayed recall), mean (SD)	8.1 (2.7)	8.4 (2.7)	8.1 (2.5)
SDMT, mean (SD)	39.2 (8.8)	40.1 (8.1)	40.4 (8.9)
COWAT, mean (SD)	12.3 (4.6)	12.6 (4.7)	12.3 (4.3)

Abbreviations: IQR, interquartile range; BMI, body mass index; *APOE*ɛ4, apolipoprotein epsilon-4; CRAE, central retinal arteriolar equivalents; CRVE, central retinal venular equivalents; 3MS, Modified Mini-Mental State Examination; HVLT-R, Hopkins Verbal Learning Test Revised (delayed recall); SDMT, Symbol Digit Modalities Test; COWAT, Controlled Oral Word Association Task.

^a^BMI, 0.7% missing; eGFR-CKD, 2.6% missing; *APOE*ɛ4 carrier status, 4.9%

### Retinal vessel calibres

Participants in the narrower arteriolar calibre group (tertile 1) were more likely to be male and have a history of hypertension (Table 1). Those in the wider venular calibre group (tertile 3) were more likely to be current or former smokers and have diabetes.

The covariate-adjusted cross-sectional association between baseline RVCs and total, deep or periventricular WMH volumes were not statistically significant (Appendix Table S4).

### Association of baseline RVCs and cognitive trajectories

**Figure 1:**
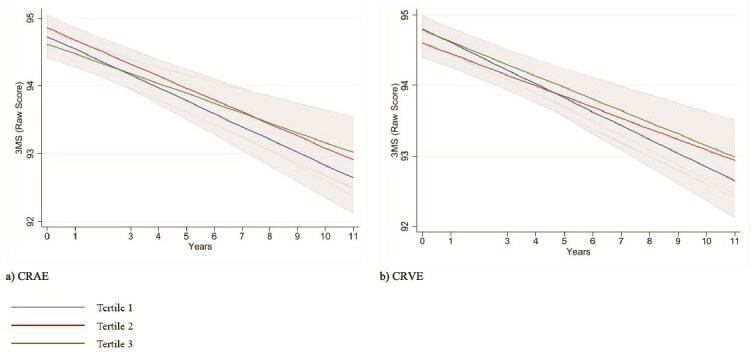
Full covariate-adjusted mean 3MS score trajectories over time by tertiles of (a) CRAE (*p-interaction* = 0.31) and (b) CRVE (*p-interaction* = 0.45). The x-axis signifies the year of cognitive assessment at baseline and years 1 to 11 (excluding year 2). The y-axis signifies raw 3MS mean scores (higher values indicate better cognition). Shaded regions indicate 95% confidence intervals. Models adjusted for age, sex, education, BMI, hypertension, dyslipidaemia, diabetes, smoking and eGFR.

Table 3 and Figure 1 present results from the fully-adjusted LMM analyses demonstrating a lack of association between baseline RVC tertiles and cognitive trajectories of up to 11-years (median = 7.4 [IQR 5.5-8.5]. The trajectories of cognitive scores over time were similar across the CRAE and CRVE tertiles in both the age, sex and education adjusted model (Appendix Table S5, Figure S3) and the fully adjusted model (Table 3) (all *p-interactions* > 0.05, Table 3). Results were congruent for trajectories in cognitive composite z-scores (Appendix Table S6).

**Table 3:** Fully adjusted mixed-effect models’ β-coefficients and 95% confidence intervals of the association between retinal vessel calibre, white matter hyperintensity volume and annual changes up to 11 yrs (CRAE × time; CRVE × time) and 7 years (WMH × time) in raw cognitive scores

β-coefficient (95% CI)^a^				
Retinal Vessel Calibres	3MS	HVLT	SDMT	COWAT
CRAE tertiles × time, yrs				
1	−0.04 (−0.11, 0.03)	−0.01 (−0.04, 0.23)	−0.00 (−0.08, 0.08)	0.03 (−0.01, 0.07)
2	−0.03 (−0.10, 0.04)	−0.01 (−0.04, 0.02)	0.00 (−0.08, 0.08)	−0.01 (−0.05, 0.03)
3	*Reference category*	*Reference category*	*Reference category*	*Reference category*
*p-interaction*	0.38	0.50	0.91	0.72
CRAE SD (15.32) × time, yrs	0.02 (−0.01, 0.05)	−0.00 (−0.01, 0.01)	0.00 (−0.03, 0.04)	−0.02 (−0.03, −0.00)
CRVE tertiles × time, yrs				
1	*Reference category*	*Reference category*	*Reference category*	*Reference category*
2	0.04 (−0.03, 0.11)	0.01 (−0.03, 0.03)	0.07 (−0.01, 0.15)	−0.03 (−0.07, −0.00)
3	0.03 (−0.04, 0.10)	0.01 (−0.03, 0.04)	0.07 (−0.01, 0.15)	−0.02 (−0.06, 0.02)
*p-interaction*	0.44	0.91	0.15	0.27
				
CRVE SD (21.92) × time, yrs	−0.06 (−0.18, 0.06)	−0.00 (−0.01, 0.01)	0.02 (−0.01, 0.05)	−0.01 (−0.03, 0.01)
White Matter Hyperintensities^b^	3MS	HVLT	SDMT	COWAT
Total WMH Tertiles × time, yrs				
1	*Reference category*	*Reference category*	*Reference category*	*Reference category*
2	−0.07 (−0.25, 0.11)	0.00 (−0.09, 0.09)	−0.18 (−0.40, 0.04)	0.01 (−0.12, 0.13)
3	−0.40 (−0.57, −0.22)	−0.13 (−0.22, −0.04)	−0.29 (−0.52, −0.07)	−0.09 (−0.22, 0.03)
*p-interaction*	<0.001	0.00	0.03	0.22
Total WMH SD (1.3) × time, yrs	−0.15 (−0.23, −0.08)	−0.05 (−0.09, −0.02)	−0.13 (−0.22, −0.03)	−0.02 (−0.07, 0.3)
Deep WMH Tertiles × time, yrs				
1	*Reference category*	*Reference category*	*Reference category*	*Reference category*
2	0.00 (−0.17, 0.18)	0.00 (−0.08, 0.09)	−0.10 (−0.33, 0.12)	−0.04 (−0.17, 0.09)
3	−0.32 (−0.50, −0.14)	−0.05 (−0.14, 0.04)	−0.20 (−0.42, 0.03)	−0.00 (−0.13, 0.12)
*p-interaction*	<0.001	0.37	0.23	0.80
Deep WMH SD (2.2) × time, yrs	−0.09 (−0.17, −0.02)	−0.03 (−0.07, 0.00)	−0.08 (−0.17, 0.01)	−0.02 (−0.07, 0.04)
Periventricular WMH Tertiles × time, yrs				
1	*Reference category*	*Reference category*	*Reference category*	*Reference category*
2	−0.12 (−0.30, 0.06)	−0.02 (−0.11, 0.06)	−0.18 (−0.40, 0.04)	−0.01 (−0.13, 0.12)
3	−0.36 (−0.54, −0.18)	−0.14 (−0.23, −0.05)	−0.29 (−0.52, −0.07)	−0.08 (−0.21, 0.05)
*p-interaction*	<0.001	0.00	0.04	0.40
Peri. WMH SD (1.2) × time, yrs	−0.15 (−0.22, −0.07)	−0.05 (−0.09, −0.02)	−0.11 (−0.21, −0.02)	−0.03 (−0.08, 0.02)

Adjusted for age, sex, education, BMI, hypertension, dyslipidaemia, diabetes, smoking, eGFR

Abbreviations: CRAE, Central Retinal Arteriolar Equivalents; CRVE, Central Retinal Venular Equivalents; WMH, White Matter Hyperintensities; yrs, years; Peri., Periventricular; 3MS, Modified Mini-Mental State Examination (score 0-100); HVLT, Hopkins Verbal Learning Test-Revised delayed recall (score 0-12); SDMT, Symbol Digit Modalities Test (score 0-133); COWAT, Controlled Oral Word Association Test (score 0-110); CI, confidence intervals.

^a^Beta-coefficients here represent the difference in the mean change in cognitive scores per year (retinal vessel calibre x time) relative to the retinal vessel calibre/white matter hyperintensity tertile reference category.

^b^Model additionally adjusted for total brain volume (minus ventricles).

### White matter hyperintensity volumes

Table 2 presents participant characteristics stratified by tertiles of total WMH volumes. Participants with larger WMH volumes were more likely to be older in age, have lower eGFR levels and possess other cardiovascular risk factors including current/former smoking, hypertension, diabetes and being an *APOE*ɛ-4 carrier.

### Association of baseline WMH volumes and cognitive trajectories

**Figure 2:**
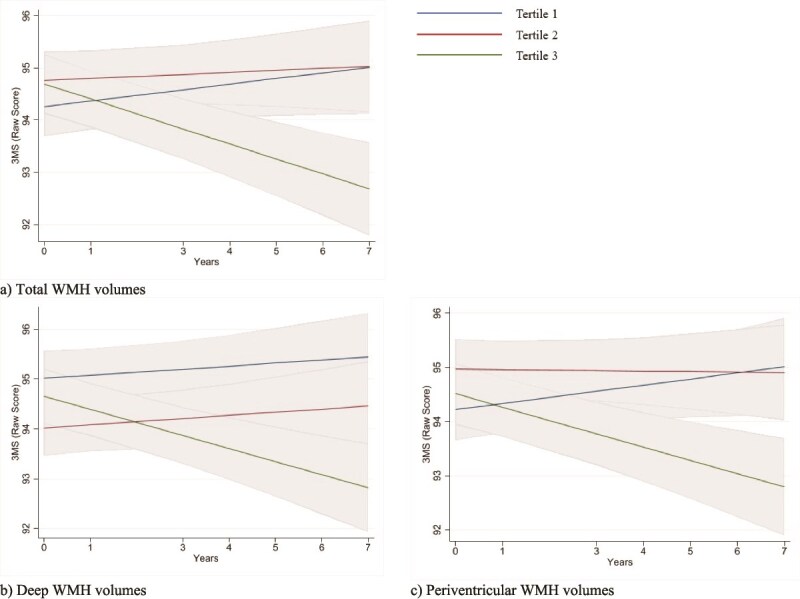
Full covariate-adjusted mean raw 3MS score trajectories over time by tertiles of (a) Total WMH volumes (*p-interaction* < 0.001); (b) Deep WMH volumes (*p-interaction* < 0.001); and (c) Periventricular WMH volumes (*p-interaction* < 0.001). The x-axis signifies the year of cognitive assessment at baseline and years 1 to 7 (excluding year 2). The y-axis signifies raw mean 3MS scores. Shaded regions indicate 95% confidence intervals. Models adjusted for age, sex, education, BMI, hypertension, dyslipidaemia, diabetes, smoking, eGFR and total brain volume.

Table 3 and Figure 2 present the fully adjusted association between baseline WMH volumes and cognitive trajectories for up to 7-years (median = 3.8 IQR 2.9, 5.3). Relative to participants in the lowest WMH volume tertile, participants in the highest tertile had more rapid declines in 3MS scores for total (β = −0.40, 95% CI −0.57, −0.22), deep (β = −0.32, 95% CI −0.50, −0.14) and periventricular (β = −0.36, 95% CI −0.54, −0.18) regions. Likewise, in the fully-adjusted model, a 1 SD increase in total, deep and periventricular WMH volumes resulted in significant decreases in 3MS scores per year (Table 3). Results were congruent for sub-domains of psychomotor speed (SDMT) and delayed memory (HVLT-R) (Table 3) and trajectories in composite z-scores (Appendix Table S6). There was no significant association between WMH volumes and COWAT score trajectories (Appendix Figure S4). Results remained consistent across models 1 (Appendix Figure S4) and 2 (Table 3).

### Further analyses

The associations between CRAE, CRVE and WMH volumes with trajectories in the primary outcome of 3MS raw scores remained robust across various sensitivity analyses. Excluding participants who had 3MS administered by phone did not alter the results (Appendix Table S7). Similarly, adjusting for *APOE*ɛ4 carrier status did not substantially change the findings (Appendix Table S8). A complete case analysis, including only participants with visits up to year 7, yielded similar results with smaller effect sizes (Appendix Table S9). Re-analysis of the entire cohort, including those who withdrew or died before completing three follow-up visits, also showed consistent results (Appendix Table S9). The IPW-adjusted models yielded results consistent with the primary unweighted analysis (Appendix Table S10 and S11).

## Discussion

In this prospective study of relatively healthy older adults, there was no association between RVC at study entry and subsequent cognitive decline over a median of 7.4 years [IQR 5.5-8.5]. In contrast, baseline total, deep and periventricular WMH volumes were significantly associated with global cognitive decline, as well as with psychomotor speed and delayed recall over a median of 3.9 [2.9-5.3] years. The results suggest that RVC is not meaningfully related to cerebrovascular processes driving cognitive decline in this population.

Our results contrast with a Singaporean cohort study using deep learning-derived RVC metrics [12], which reported significant associations between RVCs and cognitive decline and dementia However, their cohort included individuals with mild cognitive impairment and AD at baseline, unlike our cognitively healthy, population-based sample. This raises the possibility that association between RVCs may only emerge at more advanced disease stages where neurodegeneration is further pronounced. Additionally, the deep learning method employed may have greater sensitivity to detect subtle vascular alterations not captured by conventional measurement techniques. Future research could adapt these algorithms for use in non-Asian populations to assess their broader applicability.

Three large population-based cohorts including the MESA study [14], the Cardiovascular Health Study [15] and the ARIC study [13], also report no association between baseline RVCs and changes in cognitive outcomes. Despite some methodological differences (Appendix Table S1), including among either middle-aged populations or those with cardiovascular disease, these studies share key similarities with ASPREE, including the same semi-automated approach to derive CRAE and CRVE and inclusion of cognitively intact, community-dwelling participants.

This study furthermore showed no significant association between RVC and WMH volumes. While earlier studies have reported associations between specific retinal pathologies, such as diabetic or hypertensive retinopathy, and CSVD [35, 36], evidence on the association between RVCs and WMH volumes is limited. The Lothian Birth Cohort, Mild Stroke Study and MESA found no association [36, 37], whereas the Rotterdam Study found a positive association between wider venular calibres and WMH volumes [38]. Further prospective research into the link between retinal biomarkers and trajectories of brain makers of CSVD is needed to resolve these discrepant findings.

Several additional factors may explain the lack of association between RVCs and cognitive trajectories observed in our study. First, the ASPREE cohort was healthy at baseline, potentially limiting variability in both microvascular pathology and cognitive decline. RVCs may also reflect broader systemic vascular processes rather than the CSVD specifically implicated in cognitive ageing. Moreover, RVC may not be sufficiently sensitive to detect early cerebrovascular changes relevant to cognition, particularly when measured at a single baseline time-point. Emerging retinal biomarkers and novel analytic methods, including artificial intelligence, may offer greater sensitivity to microvascular pathology and improve specificity in identifying early cognitive decline [8]. Finally, while we observed no association between RVC and WMH burden in this sample, it remains possible that a longer follow-up period is required to detect downstream cognitive effects in individuals with early microvascular changes.

In contrast to the RVC findings, we found a significant association between greater baseline total, periventricular and deep WMH volumes and subsequent cognitive decline. Effect sizes for the association of periventricular WMH volumes and cognitive trajectories were marginally stronger, compared to deep regions, congruent with the extensive literature showing that periventricular WMHs are most strongly associated with declining cognitive performance [4]. This may be particularly relevant in healthy older adults, where periventricular WMHs are more prevalent and more strongly predict future cognitive decline than deep WMHs, possibly due to their earlier emergence in the disease process and their proximity to long-range white matter tracts that support executive and integrative cognitive functions [27]. Both WMH volumes and cognitive decline share known risk factors including age, lower education, female sex, *APOE*-ɛ4 allele, kidney health and cardiovascular risk factors. However, the association persisted after statistical adjustment for these potential sources of confounding.

WMHs are well-established markers of CSVD and are consistently associated with cognitive decline. The well-documented link between WMHs and cognitive performance [4] underscores the validity of these findings, which serves as a positive control for this analysis [39, 40]. Their prognostic value is supported across diverse populations, and they are increasingly considered in risk models for cognitive impairment and dementia. In contrast, RVCs may have limited standalone prognostic value in late life. Nonetheless, future research should explore whether longitudinal monitoring of retinal vessel changes, rather than single time-point assessments, could offer more sensitive indicators of cerebrovascular ageing. Combining retinal imaging with other modalities such as MRI, blood-based biomarkers and genetic risk scores may improve early detection of individuals at heightened risk of cognitive decline.

To our knowledge, this is the first study to directly compare the association of RVCs versus WMHs, a well-established CSVD marker, with cognitive decline in a healthy, older adult population. Conducted within the ASPREE cohort of older individuals free from overt cardiovascular disease, our findings contribute uniquely to the field by clarifying the limited potential for prognostic utility of RVCs relative to WMHs in late life. This offers important insight into the specificity and sensitivity of candidate biomarkers for CSVD in ageing populations.

The strengths of this study include the large population size, comprised exclusively of community-dwelling older individuals, comprehensive baseline data, longitudinal cognitive assessments, 3 T-MRI for WMH quantification and validated RVC measurements. Our data is consistent with previous reports linking narrower retinal arterioles to an increased prevalence of hypertension and wider retinal venules and arterioles with type 2 diabetes, reinforcing the validity of the methodologies used to generate the RVC data [41]. Results remained consistent following various sensitivity analyses addressing losses to follow-up, potential survivor bias, excluding participants who were administered the phone adaptation of the 3MS, after adjustment for the *APOEɛ*-4 allele carrier status and while accounting for differences in sub-group characteristics using IPW-analysis.

Limitations included reliance on a single RVC measurement at baseline, leaving open the possibility that longitudinal change in calibres may offer improved predictive capabilities. Healthy participants with no cognitive impairment and few risk factors may exhibit subtle deficits, such as impaired divided attention and altered working memory (particularly in dual-task conditions), which were not fully captured by the cognitive tests employed in this study. While significant associations between WMH burden and cognitive decline were observed, the WMH subsample had a shorter median follow-up (3.8 years), which may underestimate the long-term effects of WMH on cognition. Furthermore, this study included a subset of the broader ASPREE cohort, which may raise concerns around selection bias (Appendix Table S3). Nonetheless, results from the IPW-analysis indicate that any subtle differences in baseline characteristics between the sub-sets did not alter the overall results. Finally, our cohort was largely Caucasian, reasonably healthy at baseline and with good access to medical care, which limits the generalisability of our findings.

In this large, prospective study of healthy older adults, baseline RVC was not associated with cognitive trajectories over 11 years. In contrast, greater WMH volumes were significantly associated with global and domain-specific cognitive decline over 7 years. These findings suggest that a single measurement of RVC may not provide information relevant to future cognitive decline in initially healthy older adults.

## Supplementary Material

aa-25-0294-File002_afaf243

## Data Availability

Anonymized study data can be made available by request, to approved researchers, through the ASPREE Access Management Site: https://ams.aspree.org/public/request-data/access-aspree-data/.
