# Supplementary material for: The association of retinal vessel calibre, white matter hyperintensities and cognitive decline in community-dwelling older adults
Source: Age Ageing. 2025 Sep 10;54(9):afaf243. doi: 10.1093/ageing/afaf243 (PMC12423393; doi:10.1093/ageing/afaf243)
Supplement: aa-25-0294-File002_afaf243 [file aa-25-0294-file002_afaf243.docx]

**The association of retinal vessel calibre, white matter hyperintensities, and cognitive decline in community-dwelling older adults**

**SUPPLEMENTARY DATA**

**Supplementary Introduction and Methods**

**Table S1.** Summary of ASPREE and previous prospective cohort studies examining the association between retinal vessel calibres and longitudinal cognitive change in healthy older adults

1.1 Participants

1.2 Retinal image acquisition, processing and quality control

1.3 White matter hyperintensity volume measurement and analysis

1.4 Cognitive assessments

1.5 Definition of confounders

1.6 Data availability

**Figure S1.** Histogram of WMH data before and after log transformation

**Supplementary figures and tables**

**Figure S2**. Participant inclusion flow chart

**Table S2.** Number of participants who completed 3MS assessments at each follow-up visit in person and via phone

**Table S3.** Baseline characteristics in the ASPREE cohort, 3T MRI WMH imaging dataset, and Retinal imaging dataset

**Table S4.** The cross-sectional association between retinal vessel calibres and log-transformed WMH volumes

**Table S5.** Association between retinal vessel calibre, white matter hyperintensity volume and annual changes over 11 yrs (CRAE × time; CRVE × time) and 7 yrs (WMH × time) in cognitive scores

**Figure S3.** Marginal mean plots of HVLT-R, SDMT and COWAT score trajectories over time among CRAE and CRVE tertiles

**Figure S4.** Marginal mean plots of HVLT-R, SDMT and COWAT score trajectories over time among WMH tertiles

**Table S6.** Association between retinal vessel calibre, white matter hyperintensity volume and annual changes over 11 yrs (CRAE × time; CRVE × time) and 7 yrs (WMH × time) in cognitive composite z-scores

**Table S7.** Association between retinal vessel calibre, white matter hyperintensity volume and annual changes over 11 yrs (CRAE × time; CRVE × time) and 7 yrs (WMH × time) in global cognitive 3MS scores after exclusion of those who completed over phone administration of the 3MS

**Table S8.** Association between retinal vessel calibre, white matter hyperintensity volume and annual changes over 11 yrs (CRAE × time; CRVE × time) and 7 yrs (WMH × time) in global cognitive 3MS scores after additional adjustment for APOEɛ4 carrier status

**Table S9.** Association between retinal vessel calibre, white matter hyperintensity volume and annual changes over 7 yrs (CRAE × time; CRVE × time) and (WMH × time) in global cognitive 3MS scores Baseline characteristics in the ASPREE cohort, 3T MRI WMH imaging dataset, and Retinal imaging dataset

**Table S10.** Baseline characteristics of the retinal vessel calibre (RVC) sample weighted using inverse probability weights to match the white matter hyperintensity (WMH) subsample

**Table S11.** Linear mixed-effects model estimates (β-coefficients and 95% CIs) for the association between retinal vessel calibre and annual change in global cognition (3MS) over 11 years, using inverse probability weighting

**Introduction and Methods**

**Table S1.** Summary of ASPREE and previous prospective cohort studies examining the association between retinal vessel calibres and longitudinal cognitive change in healthy older

| **Author (year)** | **Study population (location, cognitive status, race)** | **Follow-up (years), sample (n), age (mean)** | **RVC grading method** | **Cognitive Outcomes** | **Result summary** |
| --- | --- | --- | --- | --- | --- |
| **Characteristics of the ASPREE study cohort used in these analyses** | | | | | |
| ASPREE | Australian  Cognitively healthy, no baseline cardiovascular disease  98% Caucasian | Follow-up = 11 yrs  n = 3,540  age = 73.2 | Semi-automated, IVAN | 3MS, SDMT, HVLT-R delayed recall, COWAT  Composite Scores: Executive function  Delayed memory  Global cognition | No longitudinal association |
| **Prior prospective observational studies investigating the association of RVCs and longitudinal cognitive change** | | | | | |
| Nada El Husseini et al (2024) | MESA Study, U.S. Cognitively healthy  41% Caucasian, 59% Black, Asian | Follow-up = 6.3 yrs n = 4,334 age = 61.6 | Semi-automated, IVAN | Global cognition, DSC and DS | No longitudinal association |
| Cheung et al (2022) | Singapore memory clinic cohort Cognitively healthy / MCI / dementia  100% Asian | Follow-up = 5 yrs n = 491 age = 73 (with decline) / 70 (without decline) | Fully-automated, deep-learning algorithms | CIND and dementia | Wider CRVE = increased risk CIND Narrower CRAE = increased risk CIND |
| Lesage SR et al (2009) | ARIC, U.S. Cognitively healthy  51% Caucasian, 49% Black | Follow-up = 14 yrs n = 803 age = 58 | Semi-automated, IVAN | HVLT delayed recall, SDMT, Word fluency | No longitudinal association |
| Baker ML, et al (2007) | CHS, U.S. Cognitively healthy, cardiovascular disease  85% Caucasian, 15% Black | Follow-up = 10 yrs n = 2,211 age = 78 | Semi-automated, IVAN | SDMT, MMSE, dementia | No longitudinal association |

Abbreviations: CRV, central retinal venular equivalents; CRAE, central retinal arteriolar equivalents; n, number; MESA, Multi-Ethic Study of the Elderly; ARIC, Atherosclerosis Risk in Communities; CHS, Cardiovascular Health Study; MCI, Mild Cognitively Impaired; U.S. United States; IVAN, Semi-automated Interactive Vessel Analyser software; DSC, digit symbol coding; DS, digit span; HVLT, Hopkins Verbal Learning Test; SDMT, Symbol Digit Modalities Test; CIND, cognitively impaired non-dementia

*1.1 Participants*

ASPREE was a randomised, placebo-controlled trial of daily low-dose aspirin in cognitively healthy adults aged 70+ years. Detailed methodologies and eligibility criteria have been published previously.[1-6] Briefly, Australian participants were eligible if they were 70 years of age or older and free of cardiovascular disease, physical or cognitive impairment, or any other chronic illness likely to cause death within 5-years. For those participating in MRI imaging, exclusion criteria were presence of contraindications to MRI, claustrophobia, and must have been located within a suitable geographical catchment to the Monash Biomedical Imaging facility, where MRI scans took place. There was no additional eligibility for participants who consented to receiving an eye scan.

The ASPREE and ASPREE-XT study plus relevant sub-studies, specifically those administering 3T MRI (ASPREE-NEURO)[6] and fundus photography (ASPREE-AMD and ENVISON) [4, 5] were approved by the Human Research Ethics Committees at Monash University and Alfred Hospital in Australia. All participants provided written informed consent.

*1.2 Retinal image acquisition, processing and quality control*

Participants underwent fundus photography using non-mydriatic fundus cameras and utilising Digital Health Care software (UK). Previous research had indicated that measurement from one eye enabled accurate representation of retinal vessel diameters [7]. Therefore, photographs centred on the optic disc of the right eye were analysed. In cases of missing or poor-quality right eye photographs, the left eye was used (5.6% of cases).

Automated analysis included grid placement on the optic disc, vessel type identification, and measurement of the six largest venules and arterioles from 1/2 to 1-disc diameter. Three masked graders analysed the images, with manual override capabilities and discrepancies being resolved via adjudication with a third grader. Inter-grader reliability in this sample was high (κ=0.914 - 0.969), as reflected in other study samples [8, 9].

*1.3 White matter hyperintensity volume measurement and analysis*

Relevant to this study, T1-weighted Magnetization Prepared Rapid Gradient Echo Imaging (MPRAGE, 1 mm voxels, isotropic) were acquired for brain volume and cortical thickness estimation and co-registration. Fluid-attenuated inversion recovery (FLAIR, 1.2 mm voxels) sequences were acquired for derivation of WMHs. Periventricular (≤8 mm from lateral ventricles) and deep (>8 mm from ventricles) WMH volumes were segmented using a mask of the lateral ventricles, generated from FreeSurfer brain imaging software WMH areas were derived via a 6-degrees of freedom linear transformation using the FSL Linear Registration Tool, dilated with an 8 mm box kernel using ‘fslmaths’ (FSL v.6.0.3). WMH volumes were calculated by summing all WMH voxels to yield volumes in cubic millimetres (mm³). MRI processing analysts were blinded to participant information.

*1.4 Cognitive assessments*

During the ASPREE trial, staff administered these assessments at baseline, biennially during follow-up visits (years 1, 3, 5), and at the final annual visit. In ASPREE-XT, 3MS was administered in the first year and every year thereafter, while HVLT-R, COWAT and SDMT were administered from the second year and annually thereafter. To analyse overall cognitive performance across the four cognitive tests (referred to as the "composite cognitive score")[10], the first step involved generating z-scores to standardise the results for each individual test at each time point. This was done using the formula: z = (participants score at time t – sample mean) / sample standard deviation. The z-scores for each test were then summed to calculate the composite score.

A similar approach was used to create composite scores for executive/psychomotor functioning and memory, which are key cognitive domains in older adults and assessed by multiple tests. The composite score for executive/psychomotor functioning was calculated by summing the z-scores from the SDMT, COWAT, and the similarities subscale of the 3MS. The composite memory score was derived by summing the z-scores from the HVLT-R delayed recall and the memory subscale of the 3MS.

After the COVID-19 outbreak, participants completed a validated telephone adaptation of the 3MS[11]. This adaptation excluded specific questions reducing the score range to 0-74. Scores were scaled back to 100 (scaled score = unscaled score/74 × 100) for these analyses. HVLT-R, COWAT and SDMT were not validated for phone administration, therefore, only those who completed these assessments in person were included in the analyses.

*1.5 Definition of confounders*

Extensive health, demographic, genetic, lifestyle and anthropometric data were collected as part of the ASPREE study, methods of which are reported elsewhere.[12] Baseline confounders included age at baseline (continuous), sex, education (>12-years / ≤12-years), estimated glomerular filtration rate (eGFR, continuous), body mass index (BMI, continuous), hypertension, diabetes mellitus, dyslipidaemia and smoking status (current/former vs never). Diabetes mellitus was defined by self-report of diabetes or fasting glucose ≥ 126 mg/dL or on treatment for diabetes. Hypertension was defined as SBP ≥ 140 mmHg or DBP ≥ 90 mmHg or on treatment for high blood pressure. Dyslipidemia was based on self-reported use of a statin at baseline or elevated cholesterol (either serum total cholesterol ≥212 mg/dL [≥5.5 mmol/L] or LDL >160mg/dL [>4.1 mmol/L).The apolipoprotein (*APOE*) genotype was determined directly through two single-nucleotide polymorphisms (SNPs), rs7412 and rs429358, extracted using plink v1.9.[13] The *APOE* genotype variable was then divided into those who are *APOE*ɛ4 carriers versus non-carriers.

| **Raw WMH volumes** | **Log Transformed WMH volumes** |
| --- | --- |
| Total WMH volumes | |
| 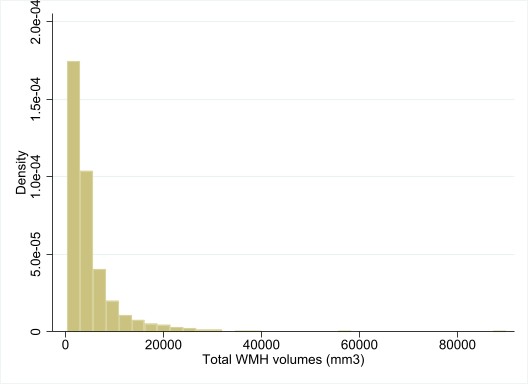 | 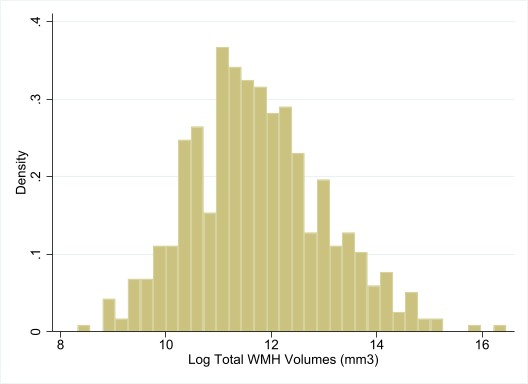 |
| Deep WMH volumes | |
| 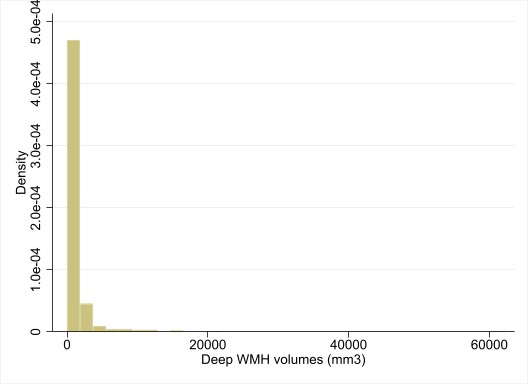 | 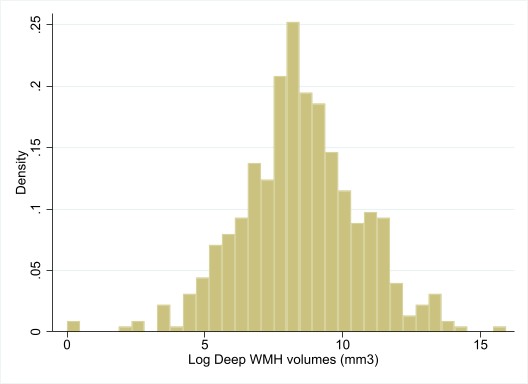 |
| Periventricular WMH volumes | |
| 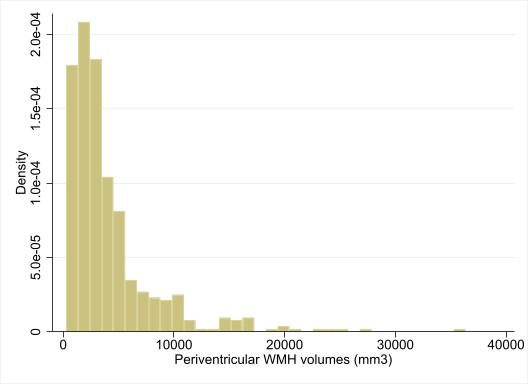 | 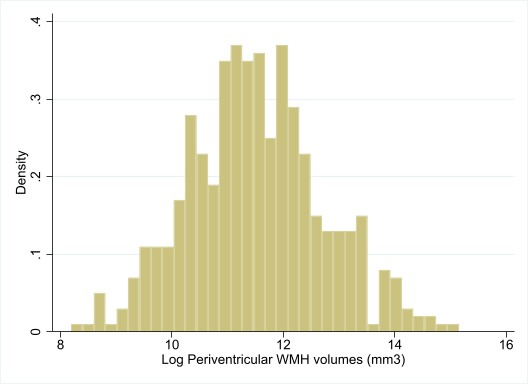 |

**Figure S1.** Histogram of WMH raw data before and after log transformation

**Supplementary figures and tables**

**
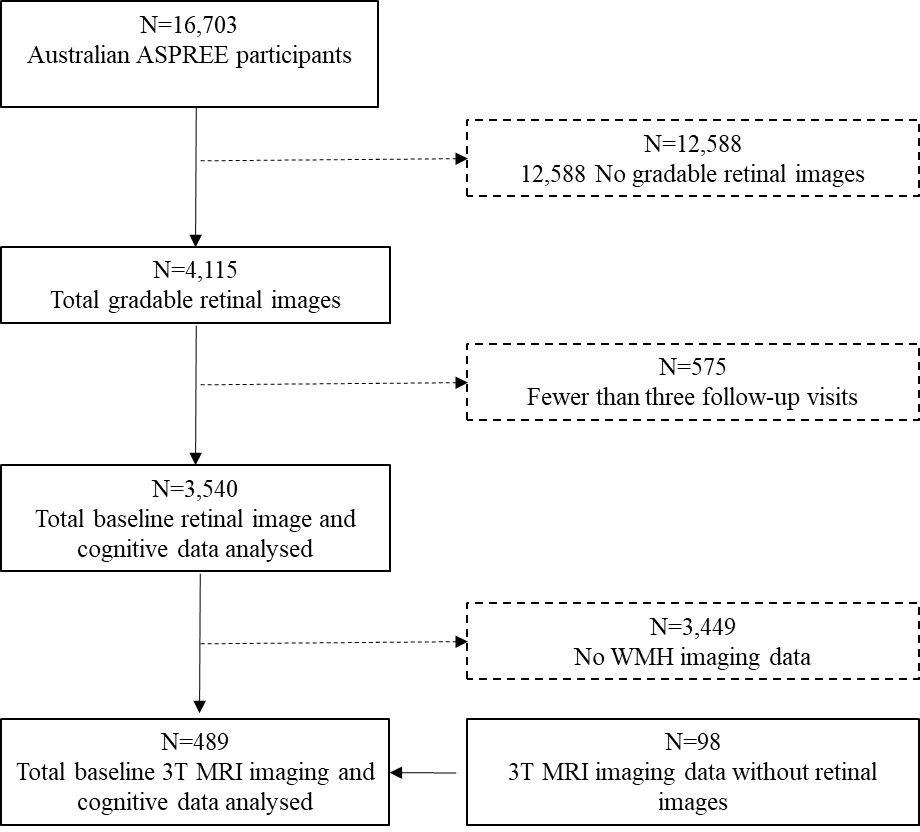
**

**Figure S2.** Participant inclusion flow chart

**Table S2.** Number of participants who completed 3MS assessments and mean 3MS (SD) raw scores at each follow-up visit in person

| **Visit** | **RVC** | **Mean (standard deviation) 3MS scores** | | | | | | **WMH** | **Mean (standard deviation) 3MS scores** | | |
| --- | --- | --- | --- | --- | --- | --- | --- | --- | --- | --- | --- |
|  | **n (% phone administered)** | **CRAE** | | | **CRVE** | | | **n (% phone administered)** | **WMH volumes (mm^3^)** | | |
|  |  | **T1** | **T2** | **T3** | **T1** | **T2** | **T3** |  | **T1** | **T2** | **T3** |
| Baseline | 3,540 (0) | 94.0 (4.3) | 93.8 (4.2) | 94.1 (4.2) | 94.1 (4.2) | 93.8 (4.4 | 94.0 (4.2) | 489 (0) | 94.0 (4.6) | 94.2 (4.1) | 93.8 (4.0) |
| Year 1 | 3,523 (0) | 94.7 (4.1) | 95.0 (4.0) | 94.9 (4.1) | 95 (4.0) | 94.8 (4.1) | 94.9 (4.0) | 488 (0) | 94.7 (4.3) | 95.3 (4.1) | 94.5 (4.7) |
| Year 2 | 2 (0) | - | - | - | - | - | - | 0 (0) | - | - | - |
| Year 3 | 3,433 (0) | 94.4 (4.8) | 94.5 (4.5) | 94.4 (4.4) | 94.5 (4.7 | 94.2 (4.8) | 94.6 (4.2) | 482 (0) | 94.7 (4.5) | 95.4 (4.4) | 93.5 (5.2) |
| Year 4 | 2,064 (0) | 94.1 (5.7) | 94.6 (4.8) | 94.4 (5.1) | 94.4 (5.4) | 94.2 (5.5) | 94.5 (4.7) | 478 (0) | 95.1 (4.1) | 95.6 (3.8) | 93.5 (5.4) |
| Year 5 | 3,148 (2.4) | 94.1 (5.6) | 94.6 (5.1) | 94.5 (5.4) | 94.4 (5.1) | 94.2 (5.7) | 94.5 (5.3) | 427 (8.7) | 95.0 (3.9) | 95.7 (4.1) | 93.7 (7.0) |
| Year 6 | 2,795 (22.1) | 94.1 (6.0) | 94.1 (6.7) | 94.4 (5.9) | 94.0 (6.4) | 94.2 (6.1) | 94.3 (6.0) | 424 (54.0) | 95.3 (4.8) | 94.9 (5.3) | 93.1 (8.9) |
| Year 7 | 2,866 (27.4) | 94.1 (6.0) | 93.9 (6.7) | 94.3 (5.9) | 94.2 (6.4 | 94.0 (6.3) | 94.0 (6.2) | 424 (38.4) | 94.9 (4.9) | 95.3 (4.9) | 93.4 (7.5) |
| Year 8 | 1,643 (35.2) | 94.3 (6.3) | 93.5 (6.7) | 93.7 (7.2) | 94.2 (6.0) | 93.8 (7.3) | 93.5 (7.0) | 36 (19.4) | - | - | - |
| Year 9 | 972 (43.0) | 94.0 (6.6) | 93.2 (6.8) | 94.4 (5.4) | 93.8 (6.8) | 94.0 (6.2) | 93.6 (6.0) | - | - | - | - |
| Year 10 | 633 (45.7) | 93.4 (6.7) | 92.9 (7.6) | 94.0 (6.5) | 93.3 (6.8) | 92.8 (7.7) | 94.2 (6.1) | **-** | **-** | **-** | **-** |
| Year 11 | 137 (26.3) | 93.1 (6.7) | 93.6 (6.8) | 93.2 (8.1) | 92.7 (6.6) | 94.9 (7.7) | 92.5 (7.9) | **-** | **-** | **-** | **-** |

**TABLE S3.** Baseline characteristics in the ASPREE cohort, WMH imaging dataset and retinal imaging dataset

|  | **ASPREE** | **3T-MRI WMH Dataset** | **Retinal image dataset** |
| --- | --- | --- | --- |
|  | **N=19,114** | **N=489** | **N=3,540** |
| Age at randomization (years) |  |  |  |
| Median (IQR) | 74.0 (71.6, 77.7) | 72.5 (71.2, 75.4) | 73.2 (71.4, 76.3) |
| Sex, n (%) female | 10782 (56.4) | 233 (47.6) | 1,872 (52.9) |
| Education, n (%<12-years) | 8,636 (45.2) | 186 (38.0) | 1,515 (42.8) |
| BMI (kg/m2), mean (SD) | 28.1 (4.7) | 27.9 (4.4) | 28.1 (4.5) |
| Smoking, current/former n (%) | 8,534 (44.6) | 207 (42.3) | 1,514 (42.8) |
| Diabetes, n (%) | 2045 (10.7) | 57 (11.7) | 342 (9.7) |
| Hypertension, n (%) | 14195 (74.3) | 347 (71.0) | 3,557 (72.2) |
| Dyslipidaemia, n (%) | 12467 (65.2) | 283 (57.9) | 2,334 (65.9) |
| Baseline 3MS, mean (SD) | 93.4 (4.6) | 94.0 (4.3) | 93.7 (4.3) |
| *APOE*ɛ4 carrier | 3,756 (25.8) | 117 (25.1) | 813 (24.5) |
| CRAE (µm), mean (SD) | - | 145.9 (15.7) | 142.3 (15.3) |
| CRVE (µm), mean (SD) | - | 212.5 (22.1) | 208.4 (21.8) |
| Total WMH volume (mm^3^), median (IQR) | - | 3,224 (1,872, 5,797) | 3,189 (1,869, 5921) |

Abbreviations: N, number; SD, standard deviation; BMI, body mass index; DBP, diastolic blood pressure; SBD, systolic blood pressure; HDL, high-density lipoprotein; eGFR CKD, estimated glomerular filtration rate (chronic kidney disease).

**TABLE S4.** Linear regression models’ β-coefficients and 95% confidence intervals (95% CIs) of the cross-sectional association between retinal vessel calibres and log-transformed WMH volumes

|  | **β-coefficient (95% CI)^a^** | |  |
| --- | --- | --- | --- |
|  | **Total** | **Periventricular** | **Deep** |
| **CRAE Tertiles** |  |  |  |
| 1 | 0.00 (-0.22, 0.23) | -0.00 (-0.23, 0.22) | -0.01 (-0.25, 0.22) |
| 2 | 0.06 (-0.16, 0.27) | 0.03 (-0.19, 0.26) | 0.16 (-0.06, 0.41) |
| 3 | *Reference category* | *Reference category* | *Reference category* |
| **CRVE Tertiles** |  |  |  |
| 1 | *Reference category* | *Reference category* | *Reference category* |
| 2 | 0.05 (-0.19, 0.29) | 0.02 (-0.23, 0.26) | 0.05 (-0.20, 0.31) |
| 3 | 0.13 (-0.09, 0.35) | 0.11 (-0.11, 0.34) | 0.17 (-0.07, 0.40) |

Controlled for age and sex, education, total brain volume, BMI, hypertension, dyslipidaemia, diabetes, smoking, eGFR

**TABLE S5.** Mixed-effect models’ β-coefficients and 95% confidence intervals (95% CIs) of the association between retinal vessel calibre, white matter hyperintensity volumes, and annual changes over 11 yrs (CRAE × time; CRVE × time) and 7 yrs (WMH × time) in cognitive scores

|  | **β-coefficient (95% CI)^a^** | | | |
| --- | --- | --- | --- | --- |
| Retinal Vessel Calibres | 3MS | HVLT-R | SDMT | COWAT |
| CRAE tertiles × time, yrs |  |  |  |  |
| 1 | -0.04 (-0.11, 0.03) | -0.01 (-0.04, 0.02) | 0.00 (-0.08, 0.08) | 0.03 (-0.01, 0.07) |
| 2 | -0.03 (-0.10, 0.04) | -0.01 (-0.04, 0.02) | 0.01 (-0.07, 0.09) | -0.01 (-0.05, 0.03) |
| 3 | *Reference category* | *Reference category* | *Reference category* | *Reference category* |
| *p-interaction* | 0.43 | 0.60 | 0.79 | 0.74 |
|  |  |  |  |  |
| CRAE SD (15.32) × time, yrs | 0.02 (-0.01, 0.05) | 0.00 (-0.01, 0.01) | 0.00 (-0.03, 0.03) | -0.02 (-0.03, -0.00) |
|  |  |  |  |  |
| CRVE tertiles × time, yrs |  |  |  |  |
| 1 | *Reference category* | *Reference category* | *Reference category* | *Reference category* |
| 2 | 0.03 (-0.03, 0.10) | 0.01 (-0.02, 0.04) | 0.07 (-0.01, 0.15) | -0.04 (-0.08, 0.00) |
| 3 | 0.03 (-0.04, 0.10) | 0.01 (-0.02, 0.04) | 0.07 (-0.01, 0.15) | -0.02 (-0.06, 0.02) |
| *p-interaction* | 0.52 | 0.84 | 0.13 | 0.21 |
|  |  |  |  |  |
| CRVE SD (21.92) × time, yrs | 0.01 (-0.01, 0.04) | 0.00 (-0.01, 0.01) | 0.02 (-0.01, 0.05) | -0.01 (-0.03, 0.00) |
| White Matter Hyperintensities | 3MS^b^ | HVLT-R^b^ | SDMT^b^ | COWAT^b^ |
| Total WMH^c^ Tertiles × time, yrs |  |  |  |  |
| 1 | *Reference category* | *Reference category* | *Reference category* | *Reference category* |
| 2 | -0.06 (-0.24, 0.11) | 0.00 (-0.08, 0.09) | -0.19 (-0.41, 0.02) | 0.01 (-0.11, 0.13) |
| 3 | -0.41 (-0.58, -0.23) | -0.14 (-0.22, -0.05) | -0.29 (-0.52, -0.07) | -0.10 (-0.22, 0.03) |
| *p-interaction* | <0.001 | 0.00 | 0.03 | 0.20 |
|  |  |  |  |  |
| Total WMH SD (1.3) × time, yrs | -0.16 (-0.23, -0.09) | -0.05 (-0.09, -0.02) | -0.13 (-0.22, -0.04) | -0.02 (-0.07, 0.03) |
|  |  |  |  |  |
| Deep WMH^c^ Tertiles × time, yrs |  |  |  |  |
| 1 | *Reference category* | *Reference category* | *Reference category* | *Reference category* |
| 2 | 0.01 (-0.17, 0.18) | 0.01 (-0.08, 0.09) | -0.09 (-0.31, 0.13) | -0.03 (-0.16, 0.09) |
| 3 | -0.33 (-0.51, -0.16) | -0.06 (-0.15, 0.03) | -0.19 (-0.41, 0.04) | -0.00 (-0.13, 0.12) |
| *p-interaction* | <0.001 | 0.29 | 0.26 | 0.84 |
|  |  |  |  |  |
| Deep WMH SD (2.2) × time, yrs | -0.10 (-0.18, -0.03) | -0.03 (-0.07, 0.00) | -0.08 (-0.17, 0.01) | -0.02 (-0.07, 0.04) |
|  |  |  |  |  |
| Periventricular WMH^c^ Tertiles × time, yrs |  |  |  |  |
| 1 | *Reference category* | *Reference category* | *Reference category* | *Reference category* |
| 2 | -0.12 (-0.29, 0.06) | -0.02 (-0.11 (0.07) | -0.17 (-0.39, 0.04) | -0.01 (-0.13, 0.12) |
| 3 | -0.37 (-0.54, -0.19) | -0.14 (-0.23, -0.05) | -0.31 (-0.54, -0.09) | -0.08 (-0.21, 0.04) |
| *p-interaction* | <0.001 | 0.00 | 0.02 | 0.36 |
|  |  |  |  |  |
| Peri. WMH SD (1.2) × time, yrs | -0.15 (-0.23, -0.08) | -0.05 (-0.09, -0.02) | -0.12 (-0.21, -0.03) | -0.03 (-0.08, 0.02) |

Adjusted for age, sex and education

^a^Beta-coefficients here represent the difference in the mean change in cognitive scores per year (retinal vessel calibre x time) relative to the retinal vessel calibre and WMH tertile reference category.

^b^LMMs include data from baseline, year 1, year 3, years 5-7. Other time-points have a sample of <100.

^c^Models 1 and 2 additionally adjusted for total brain volume (minus ventricles).

Abbreviations: CRAE, Central Retinal Arteriolar Equivalents; CRVE, Central Retinal Venular Equivalents.

*P-value < 0.05; ** p-value <0.01; *** p-value <0.001

| **CRAE** | **CRVE** |
| --- | --- |
| **HVLT-R** | |
| 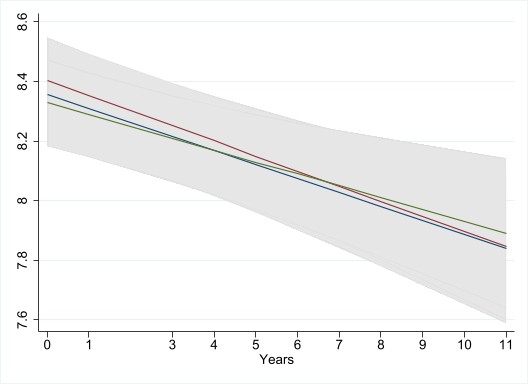 | 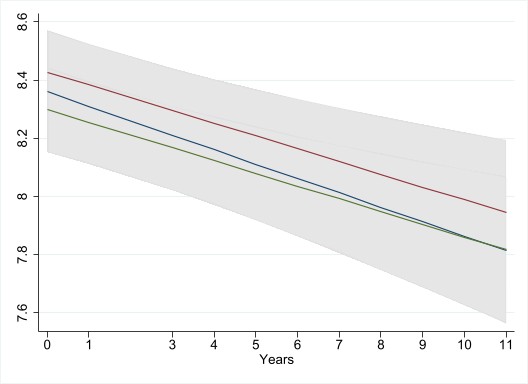 |
| **SDMT** | |
| 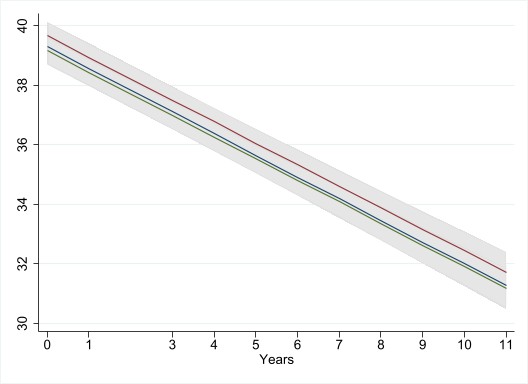 | 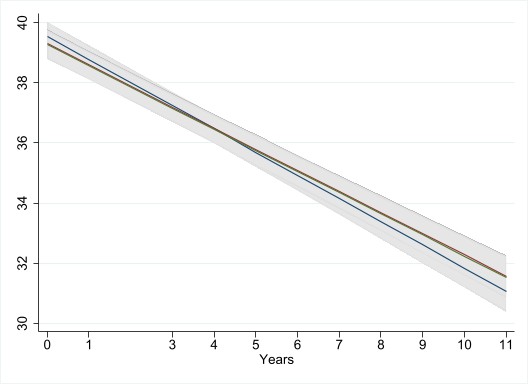 |
| **COWAT** | |
| 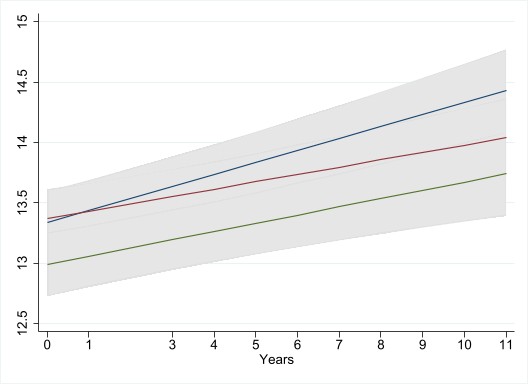 | 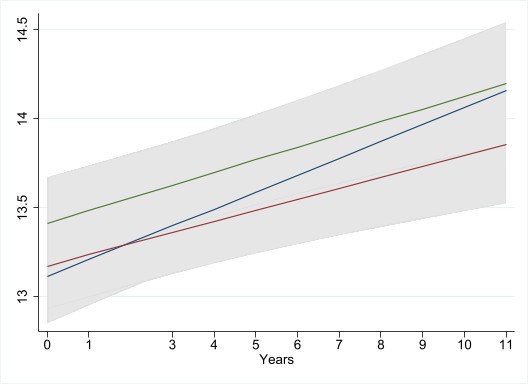 |
| 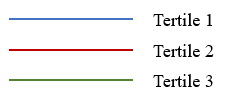 |  |

**Figure S3.** Full covariate-adjusted mean HVLT-R, SDMT and COWAT score trajectories over time by tertiles of CRAE and CRVE. The x-axis signifies the year of cognitive assessment at baseline and years 1 to 11 (excluding year 2). The y-axis signifies raw cognitive mean scores (higher values indicate better cognition). Shaded regions indicate 95% confidence intervals. Abbreviations: CRAE, Central Retinal Arteriolar Equivalents; CRVE, Central Retinal Venular Equivalents; 3MS, Modified Mini-Mental State Examination (score 0-100); HVLT, Hopkins Verbal Learning Test-Revised delayed recall (score 0-12); SDMT, Symbol Digit Modalities Test (score 0-133); COWAT, Controlled Oral Word Association Test (score 0-110); CI, confidence intervals.

.Models adjusted for age, sex, education, BMI, hypertension, dyslipidaemia, diabetes, smoking, eGFR.

| **HVLT-R** |
| --- |
| **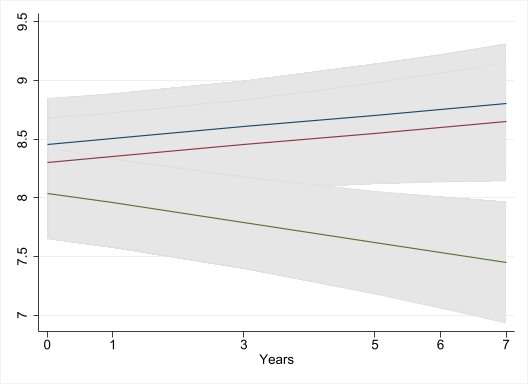** |
| **SDMT** |
| **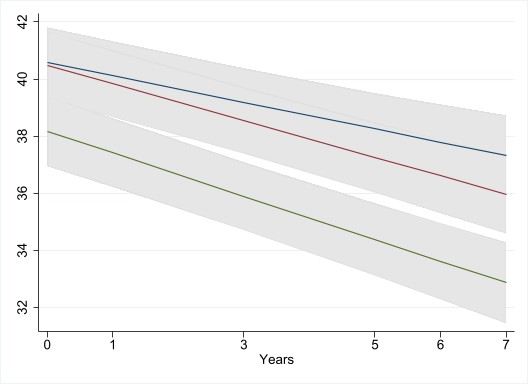** |
| **COWAT** |
| **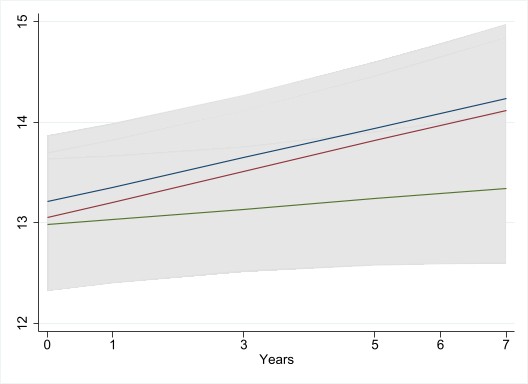** |
| 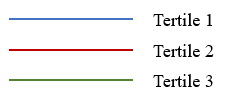 |

**Figure S4.** Full covariate-adjusted mean HVLT-R, SDMT and COWAT score trajectories over time by tertiles of Total WMH volumes. The x-axis signifies the year of cognitive assessment at baseline and years 1 to 7 (excluding year 2). The y-axis signifies raw mean cognitive scores. Shaded regions indicate 95% confidence intervals.

Abbreviations: WMHs, White Matter Hyperintensities; 3MS, Modified Mini-Mental State Examination (score 0-100); HVLT, Hopkins Verbal Learning Test-Revised delayed recall (score 0-12); SDMT, Symbol Digit Modalities Test (score 0-133); COWAT, Controlled Oral Word Association Test (score 0-110); CI, confidence intervals. Models adjusted for age, sex, education, BMI, hypertension, dyslipidaemia, diabetes, smoking, eGFR and total brain volume.

**TABLE S6.** Mixed-effect models’ β-coefficients (95% CI’s) of the association between retinal vessel calibre, white matter hyperintensity volume and annual changes over 11 yrs (CRAE × time; CRVE × time) and 7 yrs (WMH × time) in cognitive composite z-scores

|  | β-coefficient (95% CI)^a^ | | |
| --- | --- | --- | --- |
| Retinal Vessel Calibres | Global composite | Executive composite | Memory composite |
| CRAE tertiles × time, yrs |  |  |  |
| 1 | 0.00 (-0.01, 0.01) | 0.00 (-0.00, 0.01) | -0.00 (-0.01, 0.01) |
| 2 | -0.00 (-0.01, 0.01) | 0.00 (-0.00, 0.01) | -0.00 (-0.01, 0.00) |
| 3 | *Reference category* | *Reference category* | *Reference category* |
| *p-interaction* | 0.54 | 0.28 | 0.31 |
|  |  |  |  |
| CRAE SD (15.32) × time, yrs | -0.00 (-0.00, 0.00) | -0.00 (-0.00, 0.00) | 0.00 (-0.00, 0.00) |
|  |  |  |  |
| CRVE tertiles × time, yrs |  |  |  |
| 1 | *Reference category* | *Reference category* | *Reference category* |
| 2 | 0.00 (-0.01, 0.01) | 0.00 (-0.00, 0.01) | 0.00 (-0.01, 0.01) |
| 3 | 0.00 (-0.01, 0.01) | 0.00 (-0.00, 0.01) | -0.00 (-0.01, 0.01) |
| *p-interaction* | 0.93 | 0.25 | 0.80 |
|  |  |  |  |
| CRVE SD (21.92) × time, yrs | -0.00 (-0.00, 0.00) | 0.00 (-0.00, 0.00) | 0.00 (-0.00, 0.00) |
| White Matter Hyperintensities^b^ | Global composite | Executive composite | Memory composite |
| Total WMH Tertiles × time, yrs |  |  |  |
| 1 | *Reference category* | *Reference category* | *Reference category* |
| 2 | 0.01 (-0.04, 0.01) | 0.00 (-0.01, 0.02) | -0.01 (-0.03, 0.01) |
| 3 | -0.04 (-0.06, -0.02) | -0.01 (-0.03, 0.00) | -0.03 (-0.05, -0.01) |
| *p-interaction* | 0.002 | 0.16 | 0.004 |
|  |  |  |  |
| Total WMH SD (1.3) × time, yrs | -0.02 (-0.03, -0.01) | -0.01 (-0.01, -0.00) | -0.01 (-0.02, -0.00) |
|  |  |  |  |
| Deep WMH Tertiles × time, yrs |  |  |  |
| 1 | *Reference category* | *Reference category* | *Reference category* |
| 2 | -0.01 (-0.03, 0.01) | -0.00 (-0.02, 0.02) | 0.01 (-0.01, 0.03) |
| 3 | -0.02 (-0.05, -0.00) | -0.01 (-0.02, 0.01) | -0.03 (-0.05, -0.01) |
| *p-interaction* | 0.12 | 0.75 | <0.001 |
|  |  |  |  |
| Deep WMH SD (2.2) × time, yrs | -0.01 (-0.02, -0.00) | -0.00 (-0.01, 0.00) | -0.01 (-0.02, 0.00) |
|  |  |  |  |
| Periventricular WMH Tertiles × time, yrs |  |  |  |
| 1 | *Reference category* | *Reference category* | *Reference category* |
| 2 | -0.02 (-0.02, 0.00) | -0.00 (-0.02, 0.02) | -0.02 (-0.04, 0.00) |
| 3 | -0.04 (-0.06, -0.02) | -0.02 (-0.03, 0.00) | -0.03 (-0.05, -0.01) |
| *p-interaction* | 0.003 | 0.11 | 0.02 |
|  |  |  |  |
| Peri. WMH SD (1.2) × time, yrs | -0.02 (-0.03, -0.01) | -0.01 (-0.02, -0.00) | -0.01 (-0.02, -0.00) |
|  |  |  |  |

Adjusted for age, sex, education, BMI, hypertension, dyslipidaemia, diabetes, smoking, eGFR

^a^Beta-coefficients here represent the difference in the mean change in cognitive scores per year (retinal vessel calibre x time) and WMH tertiles relative to the reference category.

^b^Model additionally adjusted for total brain volume (minus ventricles).

Abbreviations: CRAE, Central Retinal Arteriolar Equivalents; CRVE, Central Retinal Venular Equivalents; WMH, White Matter Hyperintensities; yrs, years; Peri., Periventricular.

**Further analyses**

**TABLE S7.** Mixed-effect models’ β-coefficients and 95% confidence intervals (95% CIs) of the association between retinal vessel calibre, white matter hyperintensity volume and annual changes over 11 yrs (CRAE × time; CRVE × time) and 7 yrs (WMH × time) in global cognitive 3MS scores **after exclusion of those who completed over phone administration of the 3MS**

|  | **β-coefficient (95% CI)^a^** |
| --- | --- |
| **Retinal Vessel Calibres** | **3MS** |
| CRAE tertiles × time, yrs |  |
| 1 | -0.04 (-0.11, 0.03) |
| 2 | -0.05 (-0.12, 0.02) |
| 3 | *Reference category* |
| *p-interaction* | 0.19 |
| CRVE tertiles × time, yrs |  |
| 1 | *Reference category* |
| 2 | 0.04 (-0.03, 0.11) |
| 3 | 0.01 (-0.06, 0.08) |
| *p-interaction* | 0.57 |
| **White Matter Hyperintensities** |  |
| Total WMH^b^ Tertiles × time, yrs |  |
| 1 | *Reference category* |
| 2 | -0.06 (-0.24, 0.11) |
| 3 | -0.38 (-0.55, -0.21) |
| *p-interaction* | <0.001 |
| Deep WMH^b^ Tertiles × time, yrs |  |
| 1 | *Reference category* |
| 2 | 0.04 (-0.13, 0.22) |
| 3 | -0.29 (-0.47, -0.12) |
| *p-interaction* | <0.001 |
| Periventricular WMH^b^ Tertiles × time, yrs |  |
| 1 | *Reference category* |
| 2 | -0.14 (-0.31, 0.03) |
| 3 | -0.33 (-0.51, -0.15) |
| *p-interaction* | <0.001 |

Adjusted for age, sex, education, BMI, hypertension, dyslipidaemia, diabetes, smoking, eGFR

^a^Beta-coefficients here represent the difference in the mean change in cognitive scores per year (exposure x time) relative to the retinal vessel calibre / WMH reference category.

^b^Models additionally adjusted for total brain volume (minus ventricles).

Abbreviations: CRAE, Central Retinal Arteriolar Equivalents; CRVE, Central Venular Equivalents; WMH, White Matter Hyperintensities.

**TABLE S****8.** Mixed-effect models’ β-coefficients and 95% confidence intervals (CIs) of the association between retinal vessel calibre, white matter hyperintensity volume and annual changes over 11 yrs (CRAE × time; CRVE × time) and 7 yrs (WMH × time) in global cognitive 3MS scores **after additional adjustment for *APOEɛ4* carrier status**

|  | **β-coefficient (95% CI)^a^** |
| --- | --- |
| **Retinal Vessel Calibres** | **3MS** |
| CRAE tertiles × time, yrs |  |
| 1 | -0.05 (-0.12, 0.02) |
| 2 | -0.04 (-0.11, 0.03) |
| 3 | *Reference category* |
| *p-interaction* | 0.30 |
| CRVE tertiles × time, yrs |  |
| 1 | *Reference category* |
| 2 | 0.06 (-0.01, 0.13) |
| 3 | 0.05 (-0.02, 0.12) |
| *p-interaction* | 0.25 |
|  |  |
| **White Matter Hyperintensities** |  |
| Total WMH^b^ Tertiles × time, yrs |  |
| 1 | *Reference category* |
| 2 | -0.07 (-0.25, 0.11) |
| 3 | -0.37 (-0.55, -0.19) |
| *p-interaction* | <0.001 |
| Deep WMH^b^ Tertiles × time, yrs |  |
| 1 | *Reference category* |
| 2 | 0.00 (-0.18, 0.18) |
| 3 | -0.29 (-0.47, -0.12) |
| *p-interaction* | <0.001 |
| Periventricular WMH^b^ Tertiles × time, yrs |  |
| 1 | *Reference category* |
| 2 | -0.12 (-0.30, 0.06) |
| 3 | -0.33 (-0.51, -0.15) |
| *p-interaction* | <0.001 |

Adjusted for age, sex, education, BMI, hypertension, dyslipidaemia, diabetes, smoking, eGFR and *APOE*ɛ4 carrier status (carrier vs non-carrier).

^a^Beta-coefficients here represent the difference in the mean change in cognitive scores per year (exposure x time) relative to the retinal vessel calibre / WMH reference category.

^b^Models additionally adjusted for total brain volume (minus ventricles).

Abbreviations: CRAE, Central Retinal Arteriolar Equivalents; CRVE, Central Venular Equivalents; WMH, White Matter Hyperintensities.

**TABLE S9.** Mixed-effect models’ β-coefficients and 95% confidence intervals (95% CIs) of the association between retinal vessel calibre, white matter hyperintensity volume and annual changes over 7 yrs (CRAE × time; CRVE × time) and (WMH × time) in global cognitive 3MS scores **among participants who had completed study visits up to year 7 and among the total cohort.**

|  | **β-coefficient (95% CI)^a^** | |
| --- | --- | --- |
|  | **7 visits completed**  **(n=2,871)** | **Total cohort**  **(n=4,115)** |
| **Retinal Vessel Calibres** | | |
| CRAE tertiles × time, yrs |  |  |
| 1 | -0.03 (-0.10, 0.03) | -0.05 (-0.12, 0.02) |
| 2 | -0.03 (-0.10, 0.03) | -0.04 (-0.11, 0.03) |
| 3 | *Reference category* | *Reference category* |
| *p-interaction* | 0.31 | 0.31 |
|  |  |  |
| CRVE tertiles × time, yrs |  |  |
| 1 | *Reference category* | *Reference category* |
| 2 | 0.02 (-0.04, 0.07) | 0.04 (-0.03, 0.11) |
| 3 | 0.00 (-0.06, 0.06) | 0.03 (-0.29, 0.35) |
| *p-interaction* | 0.71 | 0.45 |
|  |  |  |
| **White Matter Hyperintensities^b^** **7 visits completed Total cohort**  **(n=424) (n=551)** | | |
| Total WMH^b^ Tertiles × time, yrs |  |  |
| 1 | *Reference category* | *Reference category* |
| 2 | -0.07 (-0.21, 0.07) | -0.10 (-0.28, 0.07) |
| 3 | -0.23 (-0.38, -0.08)** | -0.42 (-0.60, -0.23)*** |
| *p-interaction* | 0.01 | 0.00 |
|  |  |  |
| Deep WMH^b^ Tertiles × time, yrs |  |  |
| 1 | *Reference category* | *Reference category* |
| 2 | 0.02 (-0.12, 0.16) | 0.05 (-0.13, 0.23) |
| 3 | -0.11 (-0.26, 0.04 | -0.34 (-0.52, -0.15)*** |
| *p-interaction* | 0.21 | 0.00 |
|  |  |  |
| Periventricular WMH^b^ Tertiles × time, yrs |  |  |
| 1 | *Reference category* | *Reference category* |
| 2 | -0.09 (-0.24, 0.05) | -0.12 (-0.30, 0.05) |
| 3 | -0.21 (-0.36, -0.06)** | -0.41 (-0.59, -0.22)*** |
| *p-interaction* | 0.02 | 0.00 |

Adjusted for age, sex, education, BMI, hypertension, dyslipidaemia, diabetes, smoking, eGFR

^a^Beta-coefficients here represent the difference in the mean change in cognitive scores per year (exposure x time) relative to the retinal vessel calibre / WMH reference category.

^b^Models additionally adjusted for total brain volume (minus ventricles).

Abbreviations: CRAE, Central Retinal Arteriolar Equivalents; CRVE, Central Retinal Venular Equivalents; WMH, White Matter Hyperintensities.

*P-value < 0.05; ** p-value <0.01; *** p-value <0.001

**Inverse probability weighting**

To assess and account for potential selection bias between the WMH and RVC subsamples, we conducted an inverse probability weighting (IPW) analysis based on age, sex, education, and vascular risk factors. Covariate balance between groups was assessed post-weighting and confirmed to be adequate.

The IPW-adjusted LMM model yielded results consistent with the primary analysis, supporting the robustness of our findings.

**Table S10.** Baseline characteristics of the retinal vessel calibre (RVC) sample weighted using inverse probability weights to match the white matter hyperintensity (WMH) subsample

|  | **3T-MRI WMH Dataset** | **Retinal image dataset** |
| --- | --- | --- |
|  | **N=489** | **N=3,540** |
| Age at randomization (years) |  |  |
| Median (IQR) | 72.5 (71.2-75.4) | 72.6 (71.4, 75.7) |
| Sex, % female | 47.6% | 47.6% |
| Education, %<12-years | 38.0% | 38.1% |
| Diabetes, % | 11.7% | 11.5% |
| Hypertension, % | 71.0% | 71.0% |
| Dyslipidaemia, % | 57.9% | 57.8% |

**TABLE S11.** Linear mixed-effects model estimates (β-coefficients and 95% CIs) for the association between retinal vessel calibre and annual change in global cognition (3MS) over 11 years, **using inverse probability weighting**

|  | **β-coefficient (95% CI)^a^** |
| --- | --- |
| **Retinal Vessel Calibres** | **3MS** |
| CRAE tertiles × time, yrs |  |
| 1 | -0.01 (-0.07, 0.05) |
| 2 | -0.07 (-0.13, 0.00) |
| 3 | *Reference category* |
| *p-interaction* | 0.06 |
| CRVE tertiles × time, yrs |  |
| 1 | *Reference category* |
| 2 | 0.03 (-0.03, 0.10) |
| 3 | 0.01 (-0.05, 0.08) |
| *p-interaction* | 0.65 |

Adjusted for age, sex, education, BMI, hypertension, dyslipidaemia, diabetes, smoking, eGFR

^a^Beta-coefficients here represent the difference in the mean change in cognitive scores per year (retinal vessel calibre x time) relative to the retinal vessel calibre reference category.

Abbreviations: CRAE, Central Retinal Arteriolar Equivalents; CRVE, Central Venular Equivalents; WMH, White Matter Hyperintensities.

**References:**

1. Ernst ME, Broder JC, Wolfe R, Woods RL, Nelson MR, Ryan J, et al. Health Characteristics and Aspirin Use in Participants at the Baseline of the ASPirin in Reducing Events in the Elderly - eXTension (ASPREE-XT) Observational Study. Contemp Clin Trials. 2023 Jul;130:107231.

2. Group AI. Study design of ASPirin in Reducing Events in the Elderly (ASPREE): a randomized, controlled trial. Contemp Clin Trials. 2013 Nov;36(2):555-64.

3. Parker EJ, Orchard SG, Gilbert TJ, Phung JJ, Owen AJ, Lockett T, et al. The ASPREE Healthy Ageing Biobank: Methodology and participant characteristics. PLoS One. 2024;19(2):e0294743.

4. Reid CM, Storey E, Wong TY, Woods R, Tonkin A, Wang JJ, et al. Aspirin for the prevention of cognitive decline in the elderly: rationale and design of a neuro-vascular imaging study (ENVIS-ion). BMC Neurol. 2012 Feb 8;12:3.

5. Robman L, Guymer R, Woods R, Ward S, Wolfe R, Phung J, et al. Age-related macular degeneration in a randomized controlled trial of low-dose aspirin: Rationale and study design of the ASPREE-AMD study. Contemp Clin Trials Commun. 2017 Jun;6:105-14.

6. Ward SA, Raniga P, Ferris NJ, Woods RL, Storey E, Bailey MJ, et al. ASPREE-NEURO study protocol: A randomized controlled trial to determine the effect of low-dose aspirin on cerebral microbleeds, white matter hyperintensities, cognition, and stroke in the healthy elderly. Int J Stroke. 2017 Jan;12(1):108-13.

7. Wong TY, Knudtson MD, Klein R, Klein BE, Meuer SM, Hubbard LD. Computer-assisted measurement of retinal vessel diameters in the Beaver Dam Eye Study: methodology, correlation between eyes, and effect of refractive errors. Ophthalmology. 2004 Jun;111(6):1183-90.

8. Sherry LM, Wang JJ, Rochtchina E, Wong T, Klein R, Hubbard L, et al. Reliability of computer-assisted retinal vessel measurementin a population. Clin Exp Ophthalmol. 2002 Jun;30(3):179-82.

9. Myers CE, Klein R, Knudtson MD, Lee KE, Gangnon R, Wong TY, et al. Determinants of retinal venular diameter: the Beaver Dam Eye Study. Ophthalmology. 2012 Dec;119(12):2563-71.

10. Ryan J, Storey E, Murray AM, Woods RL, Wolfe R, Reid CM, et al. Randomized placebo-controlled trial of the effects of aspirin on dementia and cognitive decline. Neurology. 2020 Jul 21;95(3):e320-e31.

11. Norton MC, Tschanz JA, Fan X, Plassman BL, Welsh-Bohmer KA, West N, et al. Telephone adaptation of the Modified Mini-Mental State Exam (3MS). The Cache County Study. Neuropsychiatry Neuropsychol Behav Neurol. 1999 Oct;12(4):270-6.

12. McNeil JJ, Woods RL, Nelson MR, Murray AM, Reid CM, Kirpach B, et al. Baseline Characteristics of Participants in the ASPREE (ASPirin in Reducing Events in the Elderly) Study. J Gerontol A Biol Sci Med Sci. 2017 Oct 12;72(11):1586-93.

13. Chang CC, Chow CC, Tellier LC, Vattikuti S, Purcell SM, Lee JJ. Second-generation PLINK: rising to the challenge of larger and richer datasets. Gigascience. 2015;4:7.
